# Pneumococcal vaccination rates in immunocompromised patients—A cohort study based on claims data from more than 200,000 patients in Germany

**DOI:** 10.1371/journal.pone.0220848

**Published:** 2019-08-08

**Authors:** Niklas Schmedt, Julia Schiffner-Rohe, Ralf Sprenger, Jochen Walker, Christof von Eiff, Dennis Häckl

**Affiliations:** 1 InGef – Institute for Applied Health Research Berlin, Berlin, Germany; 2 Pfizer Deutschland GmbH, Berlin, Germany; 3 Pfizer Pharma GmbH, Berlin, Germany; 4 WIG2 – Scientific Institute for Health Economics and Health System Research, Leipzig, Germany; ESIC Medical College & PGIMSR, INDIA

## Abstract

**Background:**

The German Committee on Vaccination recommends pneumococcal vaccination for infants, seniors 60+ years and patients at risk with defined underlying diseases. Aim of this study was to assess the pneumococcal vaccination rate (pnc-VR) in patients with certain incident inherited or acquired immunodeficiency or immunosuppression and to understand who vaccinates these patients who are particularly at high risk to develop a pneumococcal infection.

**Methods:**

We conducted a cohort study in patients aged 2 years or older, with a first episode of a “high-risk” condition between January 2013 and December 2014 based on a representative sample of German claims data. Pnc-VR was calculated as the proportion of patients receiving any pneumococcal vaccine within two years after first episode of “high-risk” condition. Further analyses cover pnc-VR stratified by high risk conditions and region, time to vaccination, and physician specialty administering the pneumococcal vaccination.

**Results:**

The study population comprised 204,088 incident “high-risk” patients (56% female). The overall pnc-VR within two years was 4.4% (95%-confidence interval: 4.3%-4.5%). Within specific high-risk conditions, we found the highest vaccination rate of 11.5% (10.1%-13.0%) among patients starting immunosuppressants with underlying rheumatoid arthritis followed by 9.9% (7.8%-12.4%) in HIV patients. Stratification by region revealed a slightly higher vaccination rate in Eastern (6.5%: 6.0%-6.9%) compared to Western Germany (4.2%: 4.1–4.3%). Median time to vaccination within the first two years in vaccinated patients was 332.5 days (Q1 142 days, Q3 528 days). The majority of patients (92.6%) got vaccinated by a general practitioner.

**Conclusion:**

Although these vulnerable patients need protection most, our study suggests that the overall pnc-VR after a first episode of a high-risk condition for pneumococcal disease is very low and vaccination is far too late. To prevent pneumococcal disease in patients at high risk, further efforts are needed to increase awareness and improve the timeliness of pneumococcal vaccination.

## Introduction

Community-acquired pneumonia (CAP) is causing high rates of hospitalizations and is associated with a high clinical and economic burden for healthcare systems in Europe [1]. For Germany, a total of approximately 280,000 hospitalized CAP cases in adults were reported in 2017 [2]. *Streptococcus pneumoniae* has consistently been identified as the most common pathogen in patients with CAP in Europe [1] and is often associated with a more severe clinical course, need for mechanical ventilation and oxygen treatment compared to non-pneumococcal pneumonia [3]. Besides infants and patients with chronic diseases, elderly are at particular risk for pneumococcal infections due to a reduced immune defense and a higher burden of diseases predisposing for infectious diseases. Among adults aged 55 years and older, approximately 50% suffer from two or more and 24% from at least five chronic medical conditions [4]. In a study by Pelton et al. [5], the rate of all-cause pneumonia was 1.7-fold and 1.8-fold elevated in children and elderly (60 years of age or older) with at least one chronic medical condition and 4.1-fold increased for immunocompromised elderly, and increased gradually with a higher number of conditions (i.e. risk-stacking). Depending on the underlying disease, risk ratios vary from 2.8 (HIV) to 6.7-fold (functional/anatomical asplenia).

Due to the increased risk of pneumococcal diseases, the German Committee on Vaccination (“Staendige Impfkommission”, STIKO) recommends pneumococcal vaccination in the elderly (60 years of age or older) since 1998 as well as in patients with underlying chronic diseases [6]. Since 2016 a sequential vaccination is recommended for children, adolescents and adults in the vulnerable group of patients with a congenital or acquired immuno-deficiency or immunosuppression (“high-risk patients”) as well as in patients with anatomical and foreign-material associated risks for pneumococcal meningitis. In addition, sequential vaccination is recommended for children aged 2–15 with chronic diseases such as chronic pulmonary or heart disease, diabetes treated with oral antidiabetics or insulin, or neurological disorders. (“at-risk patients”) [7]. Sequential vaccination is defined as vaccination with the 13-valent conjugate vaccine (PCV13), followed by the 23-valent polysaccharide vaccine (PPSV23).6–12 months later. For chronically ill patients aged 16 years and older and standard vaccination for seniors aged 60 years and older, STIKO recommends vaccination with PPSV23. In any case, due to only temporary protection, the vaccination with PPSV23 should be repeated at intervals of at least 6 years [7]. Similar to Germany, in most other European countries pneumococcal vaccination is also recommended for elderly and for patients with underlying conditions. However, the conditions as well as the vaccination schemes differ with regard to the recommended vaccination schemes, i.e. PCV13 only, PPSV23 only or sequential vaccination [8].

Results of previous studies indicate that the pneumococcal vaccination rate in Germany is low [9–11]. Data from the German Health Interview and Examination Survey indicated that only 31% of adults in the age group 65 to 79 years have ever received a pneumococcal vaccination dose [12]. So far, data on the pneumococcal vaccination rate and vaccinating physicians in patients with high-risk conditions for pneumococcal disease according to STIKO is limited, although these patients are at particular risk for pneumococcal infections. We therefore aimed to assess the pneumococcal vaccination rate in “high-risk patients” with a congenital or acquired immuno-deficiency or immunosuppression, and to understand who vaccinate these patients in Germany.

## Material and methods

### Data source

This study was conducted based on claims data from the InGef (former HRI) research database. At the time of the analysis, the database included anonymized longitudinal claims data from approximately 6.7 million Germans insured in one of 64 German statutory health insurances (SHIs) contributing data to the database. Claims data are transferred directly from health care providers to a specialized data center owned by SHIs, which provides data warehouse and IT services. In the data center (acting as a trust center), data is anonymized before being entered into the InGef research database. A sample representative for the German population in terms of age and sex covering approximately 4 million patients was used for this study. This reflects approximately five percent of the total population of Germany.

In brief, the InGef database includes the following information: demographic data (e.g., age, sex and region of residence); ambulatory services including services of the outpatient unit of a hospital with information on diagnoses, therapeutic and diagnostic procedures coded according to the doctor’s fee schedule (EBM, ‘Einheitlicher Bewertungsmaßstab’) as well as the physician specialties; hospital data including respective admission and discharge dates, the main and secondary discharge diagnoses as well as diagnostic and therapeutic procedures conducted in hospital coded according to the German procedural classification (OPS, ‘Operationen- und Prozedurenschlüssel’); drug prescription and dispensing data with the date of prescription and drug dispensation; reimbursed remedies and aids; and the costs of each healthcare sector from the perspective of the German SHIs. All diagnoses in the database are coded according the German modification of the 10th revision of the International Classification of Diseases (ICD-10 GM) [13].

Information on vaccination is also coded according to the doctor’s fee schedule (EBM). The database covers information on vaccination as ambulatory services as well as vaccination in outpatient unit of a hospital, whereas inpatient vaccination is not.

Data contributing to the InGef database are stored at a specialized data center according to §284 in combination with §70 and §71 Social Code Book (“Sozialgesetzbuch”, SGB) V. The data center is owned by SHIs and provides data warehouse services. In the data center (acting as a trust center), data with respect to individual insured members and health care providers (e.g. physicians, practices, hospitals, pharmacies) are anonymized by coarsening or by removing individual variables. Since all patient-level data in the InGef database are no longer social data according to § 67 Abs. 2 SGB X in combination with Art. 4 Nr. 1 of the General Data Protection Legislation (“Datenschutz-Grundverordnung”, DSGVO), institutional review board/ethical approval and informed consent of the patient was not required.

### Study design and population

We conducted a cohort study in patients with a first episode of a documented “high-risk” condition for pneumococcal disease in between January 2013 and December 2014 (pick-up period). “High-risk” conditions for pneumococcal disease were defined according to STIKO recommendation [7] and comprised functional or anatomic asplenia, sickle cell diseases and other hemoglobinopathies, malignant neoplasms (excluding non-melanoma skin cancer), stem cell transplantation, HIV infection, chronic renal failure, chronic severe liver diseases, use of immunosuppressants as well as other immunodeficiencies including diseases of white blood cells (S1 Table). Data from 2011 and 2012 was used to assess baseline characteristics and to exclude patients with prevalent “high-risk” condition (baseline period). Data for 2015 and 2016 was used for the assessment of the pneumococcal vaccination rate ensuring an individual minimum follow-up of at least 2 years (observational period).

Patients were eligible for inclusion in the cohort if they met all of the following inclusion criteria:

1. at least one documented episode of a “high-risk” condition for pneumococcal disease in the pick-up period,
2. continuous insurance of at least two years before the first episode of a high-risk condition (baseline period),
3. continuous insurance until December, 31^st^, 2016 (end of the study period) or death,
4. age of at least two years at index date (see below).

Patients were excluded from the analyses if they fulfilled any of the following criteria:

1. if they had at least one documented episode of a “high-risk” condition for pneumococcal disease in the baseline period or
2. a claim for pneumococcal vaccination (S1 Table) in the baseline period.

The index date was defined as the first documented episode of a “high-risk” condition at which all inclusion criteria were fulfilled. For hospital diagnoses, the admission date of the respective hospitalization was used as index date. Since the date of ambulatory diagnoses was only available on a quarterly basis in the data, the date of the first documented EBM-code by the diagnosing physician was considered as proxy. For OPS-codes and EBM-codes (e.g. dialysis), the exact documented date served as index date. As pneumococcal vaccination may be administered shortly before initiation of immunosuppressants (e.g. administration of TNF-alpha inhibitors), the index date in these patients was shifted to 30 days prior to the prescription date. Patients were followed up from the index date until December, 31^st^, 2016 (end of the study period), death or documented pneumococcal vaccination.

Pneumococcal vaccinations were assessed at the exact date in the outpatient setting based on documented EBM-codes (S1 Table). Based on these codes, it is possible to differentiate the type of pneumococcal vaccination, i.e. routine childhood vaccination in infants <2 years, patients aged 60+ years and other indications; however, it cannot be specified whether PCV13 or PPSV23 was used.

### Data analysis

#### Main analysis

The pneumococcal vaccination rate was calculated as the proportion of patients with any pneumococcal vaccination within two years after the index date. Corresponding 95%-confidence intervals (CI) were calculated assuming a binomial distribution.

In addition, the mean and median duration from the first episode of a “high-risk” condition for pneumococcal disease until the first pneumococcal vaccination was calculated in vaccinated patients. The corresponding 95%-confidence intervals for the mean duration were calculated assuming a Poisson distribution. Cumulative vaccination rates within two years after the index date were calculated on a quarterly basis to visualize time to vaccination.

Besides the distribution of the physician specialty administering the first pneumococcal vaccination, we obtained the distribution of the vaccinating physician specialty in vaccinated patients stratified by the physician specialty diagnosing the “high-risk” condition.

No national immunization program is in place in Germany and vaccination is coordinated on a federal basis. Further, due to the immunization history in the former German Democratic Republic, vaccination rates are deemed higher in these states. Therefore, all outcomes were analyzed overall as well as by region (“New Federal States” / Eastern Germany vs. “Old/ Federal States” /Western Germany without consideration of Berlin) as well as by Association of Statutory Health Insurance Physicians (“Kassenaerztliche Vereinigung”, AHIP). Further stratification criteria were age-group (2–15 years, 16–59 years and 60+ years) and documented “at-risk” condition for pneumococcal disease in the baseline period (yes/no) according to the list of indications by STIKO [7].

#### Subgroup analyses

In subgroup analyses, we analyzed patients with malignant neoplasms (excluding non-melanoma skin cancer), HIV, chronic renal failure and patients with rheumatologic or other diseases receiving immunosuppressants (S1 Table). In a further subgroup analysis, the study population was restricted to patients with a first episode of a high-risk condition for pneumococcal disease in 2013 in order to observe the cohort for 3 years.

#### Sensitivity analyses

To estimate the time to vaccination, the index date for patients diagnosed in the ambulatory setting was set to the date of the first documented EBM-code by the diagnosing physician in the “base-case scenario”. We additionally performed a sensitivity analysis using the date of the last documented EBM-code by the diagnosing physician or the date of vaccination in the respective quarter (whichever occurred first) as index date assuming a “best-case scenario”.

All analyses were conducted with SAS Enterprise Guide 7.1.

## Results

### Study population

**Fig 1:**
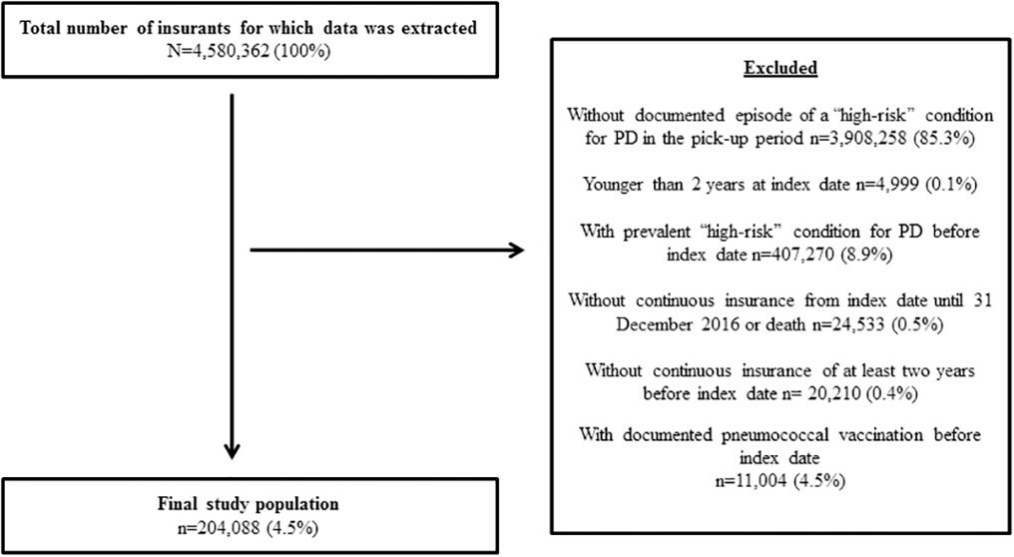
Study flow chart. PD = Pneumococcal disease.

The source population of the study comprised approximately 4.6 million subjects in the InGef database of which 204,088 patients (4.5%) with a first episode of a high-risk condition for pneumococcal disease between 2013 and 2014 were eligible for analyses (Fig 1).

The female proportion was 56.0% and most patients were in the age group 60+ years (48.0%). Most patients (52.3%) entered the cohort with a diagnosis for “other immunodeficiency” (incl. haemolytic anaemias, aplastic and other anaemias, coagulation defects, other diseases of blood and blood forming organs, certain disorders involving the immune mechanism), followed by malignant neoplasms (excl. non-melanoma skin cancer) (26.3%), and chronic renal failure (21.6%). The prevalence of “at-risk” conditions for pneumococcal disease in the baseline period in patients with a first episode of “high-risk” condition was documented with 48.3% (Table 1).

**Table 1:** Characteristics of patients with first episode of “high-risk” condition for pneumococcal disease according to STIKO.

**Total number of subjects**	204,088	(100.0%)
**“High-risk” condition at the index date** ^a^ **(n, %)**		
Functional or anatomic asplenia sickle cell diseases and other hemoglobinopathies	1,733	(0.8%)
Other immunodeficiency	106,836	(52.3%)
Malignant neoplasms excl. non-melanoma skin cancer	53,605	(26.3%)
Stem cell transplantation	8	(0.0%)
HIV infection	684	(0.3%)
Chronic renal failure	44,014	(21.6%)
Chronic severe liver disease	5,453	(2.7%)
Immunosuppressant use	5,604	(2.7%)
**“At-risk” condition in the baseline period** ^a^ **(n, %)**		
No	105,544	(51.7%)
Yes	98,544	(48.3%)
Chronic heart disease	52,299	(25.6%)
Chronic pulmonary disease	40,560	(19.9%)
Diabetes treated with oral antidiabetics or insulin	25,208	(12.4%)
Neurological disorders	26,777	(13.1%)

^a^ The same patient may be counted in several subgroups

### Pneumococcal vaccination rate within two years after first documented high-risk condition for pneumococcal disease

The pneumococcal vaccination rates within the first two years after the first documented “high-risk” condition for pneumococcal disease with corresponding 95%-CIs are displayed in Table 2. The overall pneumococcal vaccination rate with regard to administration of any pneumococcal vaccine within two years after first diagnosis of a “high-risk” condition was 4.4% (95%-CI: 4.3%-4.5%). The highest pneumococcal vaccination rate was observed in men aged 60+ years with 7.2% (7.0%-7.5%) with males showing a higher vaccination rate than females in all age groups. Within disease-specific subgroups, we found the highest vaccination rate of 11.5% (10.1%-13.0%) in patients with rheumatoid arthritis starting immunosuppressant therapy followed by 9.9% (7.8%-12.4%) in patients with HIV. Stratification by region revealed a slightly higher overall vaccination rate in Eastern Germany (6.5%: 6.0%-6.9%) compared to Western Germany (4.2%: 4.1–4.3%). Further results on the AHIP level are available in S2 Table.

**Table 2:** Pneumococcal vaccination rates with 95%-confidence intervals within two years in patients with “high-risk” condition for pneumococcal disease.

	N cohort	N vaccinated	Vaccination rate within 2 years after index date in % (95%-CI)
**Overall**	204,088	8,892	4.4 (4.3–4.5)
**Age in categories and sex**			
Women 2–15 years	4,441	31	0.7 (0.5–1.0)
Women 16–59 years	59,179	952	1.6 (1.5–1.7)
Women 60+ years	50,763	3,584	7.1 (6.8–7.3)
Men 2–15 years	4,371	42	1.0 (0.7–1.3)
Men 16–59 years	38,231	881	2.3 (2.2–2.5)
Men 60+ years	47,103	3,402	7.2 (7.0–7.5)
**“High-risk” condition at index date**			
Malignant neoplasms excl. non-melanoma skin cancer	53,605	2,490	4.7 (4.5–4.8)
HIV infection	684	68	9.9 (7.8–12.4)
Chronic renal failure	44,014	2,499	5.7 (5.5–5.9)
Immunosuppressant use with RA	1,887	217	11.5 (10.1–13.0)
Immunosuppressant use without RA	3,717	286	7.7 (6.9–8.6)
**“At-risk” condition in the baseline period**			
Yes	98,544	6,060	6.2 (6.0–6.3)
No	105,544	2,832	2.7 (2.6–2.8)
**Region**			
Old Federal States / Western Germany	188,665	7,954	4.2 (4.1–4.3)
New Federal States / Eastern Germany	12,268	791	6.5 (6.0–6.9)

CI = Confidence interval; RA = Rheumatoid arthritis

### Specialty of the physician administering pneumococcal vaccination

The large majority of patients were vaccinated by a general practitioner (92.6%). Other medical specialties rarely administered pneumococcal vaccinations, e.g. pneumologists (2.3%), rheumatologists (0.7%) or oncologists (0.3%).

Table 3 displays the distribution of the specialty of the vaccinating physician stratified by selected specialties of the physician diagnosing the “high-risk” condition at index date. Most vaccinated patients diagnosed by a general practitioner or in-hospital were vaccinated by the general practitioner with 96% and 93%, respectively. In patients diagnosed by specialists, the proportion of vaccinations by general practitioners was lower but still predominant (e.g. 76% of patients diagnosed by a rheumatologist were vaccinated by general practitioner and 20% by rheumatologists).

**Table 3:** Specialty of the vaccinating physician stratified by selected specialties of the diagnosing physician diagnosing the “high-risk” condition at index date.

Specialty of physician/setting diagnosing high-risk condition^a^	Total	General practitioner	Rheumatologist	Oncologist	In-hospital
**Total number of vaccinated subjects (n, %)**	8,892 (100%)	3,810 (100.0%)	137 (100.0%)	75 (100.0%)	2,316 (100.0%)
**Specialties of vaccinating physician (n, %)**					
General practitioner	8,232 (92.6%)	3,645 (95.7%)	104 (75.9%)	63 (84.0%)	2,158 (93.2%)
Rheumatologist	61 (0.7%)	8 (0.2%)	28 (20.4%)	<5	20 (0.9%)
Oncologist	23 (0.3%)	7 (0.2%)	<5	5 (6.7%)	<5
Pneumologist	205 (2.3%)	68 (1.8%)	<5	<5	62 (2.7%)
Other internist	86 (1.0%)	<5	<5	<5	13 (0.6%)
Pediatrician	71 (0.8%)	13 (0.3%)	<5	<5	23 (1.0%)
Other specialty	209 (2.4%)	64 (1.7%)	<5	<5	37 (1.6%)
Unknown	5 (0.1%)	<5	<5	<5	<5

^a^ Information for subgroups of less than 5 patients are not displayed due to data protection reasons

#### Time to vaccination in patients with first documented high-risk condition for pneumococcal disease

The mean and the median time to vaccination within the first two years after diagnosis of high risk condition (index date) in the 8,892 vaccinated patients was 340 days (SD [standard deviation] 219 days, 95%-confidence interval: 336 days– 345 days) and 332.5 days (Q1[1^st^ quartile] 142 days, Q3 [3^rd^ quartile] 528 days), respectively (Table 4). No meaningful differences were observed between Eastern and Western Germany. Further results on the AHIP level are available in S3 Table. After stratification, we found slight differences by age groups with the shortest mean time to vaccination for patients aged 2–15 years (291 days) and increasing time to vaccination with increasing age.

**Table 4:** Mean and median time to vaccination in days within 2 years after incident “high-risk” condition.

	Mean (SD; 95%-CI)	Median (Q1; Q3)
**Overall**	340 (219; 336 to 345)	332.5 (142; 528)
**Region**		
Western Germany	340 (220; 335 to 345)	331.5 (140; 527)
Eastern Germany	350 (216; 335 to 365)	343 (164; 537)
**Age in categories**		
2–15 years	291 (236; 236 to 346)	315 (46; 471)
16–59 years	324 (224; 314 to 334)	302 (113; 523)
60+ years	345 (218; 340 to 350)	340.5 (152–530)

CI = Confidence interval; SD = Standard deviation; Q1 = 1^st^ quartile; Q3 = 3^rd^ quartile

Sensitivity analysis assuming the best-case scenario supported our findings in mean (330 days [SD 220 days; 95% CI 325 days—334 days]) and median (322 days [Q1 133 days; Q3 517 days]) time to.

**Fig 2:**
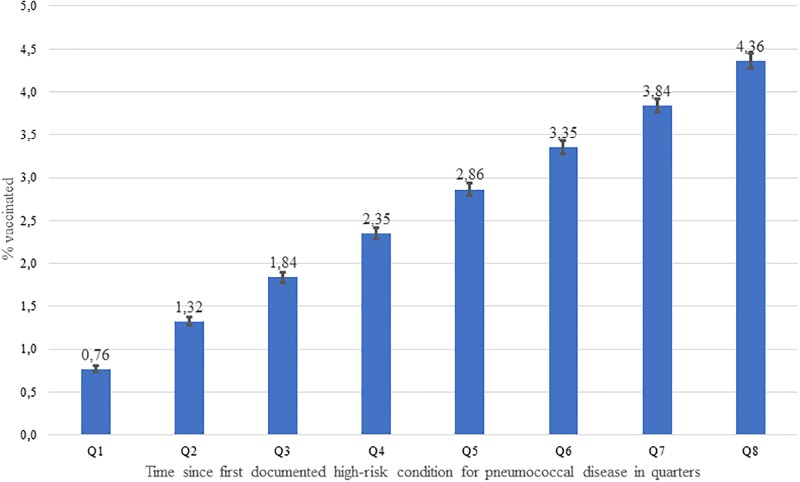
Cumulative pneumococcal vaccination rate within eight quarters after first documented “high-risk” condition for pneumococcal disease.

Fig 2 displays the cumulative pneumococcal vaccination rate within two years after the index date which increased constantly within the eight quarters after the index date.

### Subgroup analyses

Overall, 106,436 patients with an index date for high risk condition qualifying for pneumococcal vaccination in 2013 could be observed for an individual follow-up of three years. The overall pneumococcal vaccination rate was 6.2% (6.0%-6.3%) (Table 5**)**. As in the two-year follow-up, highest vaccination rates were observed in HIV patients (12.9%; 9.5%-17.0%) and in patients with rheumatoid arthritis starting immunosuppressants (12.3%; 10.4%-14.5%). Overall vaccination rates were slightly higher in “New” Federal States /Eastern Germany 9.1% (8.4%-9.8%) compared to “Old” Federal States Germany/Western Germany 6.0% (5.8%-6.1%) and in males compared to females in all age groups.

**Table 5:** Pneumococcal vaccination rates with 95%-confidence intervals within three years in patients with “high-risk” condition for pneumococcal disease.

	N cohort	N vaccinated	Vaccination rate within 3 years after index date (95%-CI)
**Overall**	106,436	6,564	6.2 (6.0–6.3)
**Age in categories and sex**			
Women 2–15 years	2,356	19	0.8 (0.5–1.3)
Women 16–59 years	30,303	676	2.2 (2.1-.2.4)
Women 60+ years	26,749	2609	9.8 (9.4–10.1)
Men 2–15 years	2,426	35	1.4 (1.0–2.0)
Men 16–59 years	19,614	632	3.2 (3.0–3.5)
Men 60+ years	24,988	2,593	10.4 (10.0–10.8)
**“High-risk” condition at the index date**			
Malignant neoplasms excl. non-melanoma skin cancer	28,187	1,908	6.8 (6.5–7.1)
HIV infection	333	43	12.9 (9.5–17.0)
Chronic renal failure	22,763	1,767	7.8 (7.4–8.1)
Immunosuppressant use with RA	1,016	125	12.3 (10.4–14.5)
Immunosuppressant use without RA	1,906	182	9.6 (8.3–11.0)
**Region**			
Old Federal States / Western Germany	98,502	5,891	6.0 (5.8–6.1)
New Federal States / Eastern Germany	6,343	576	9.1 (8.4–9.8)

CI = Confidence interval, RA = Rheumatoid arthritis

**Fig 3:**
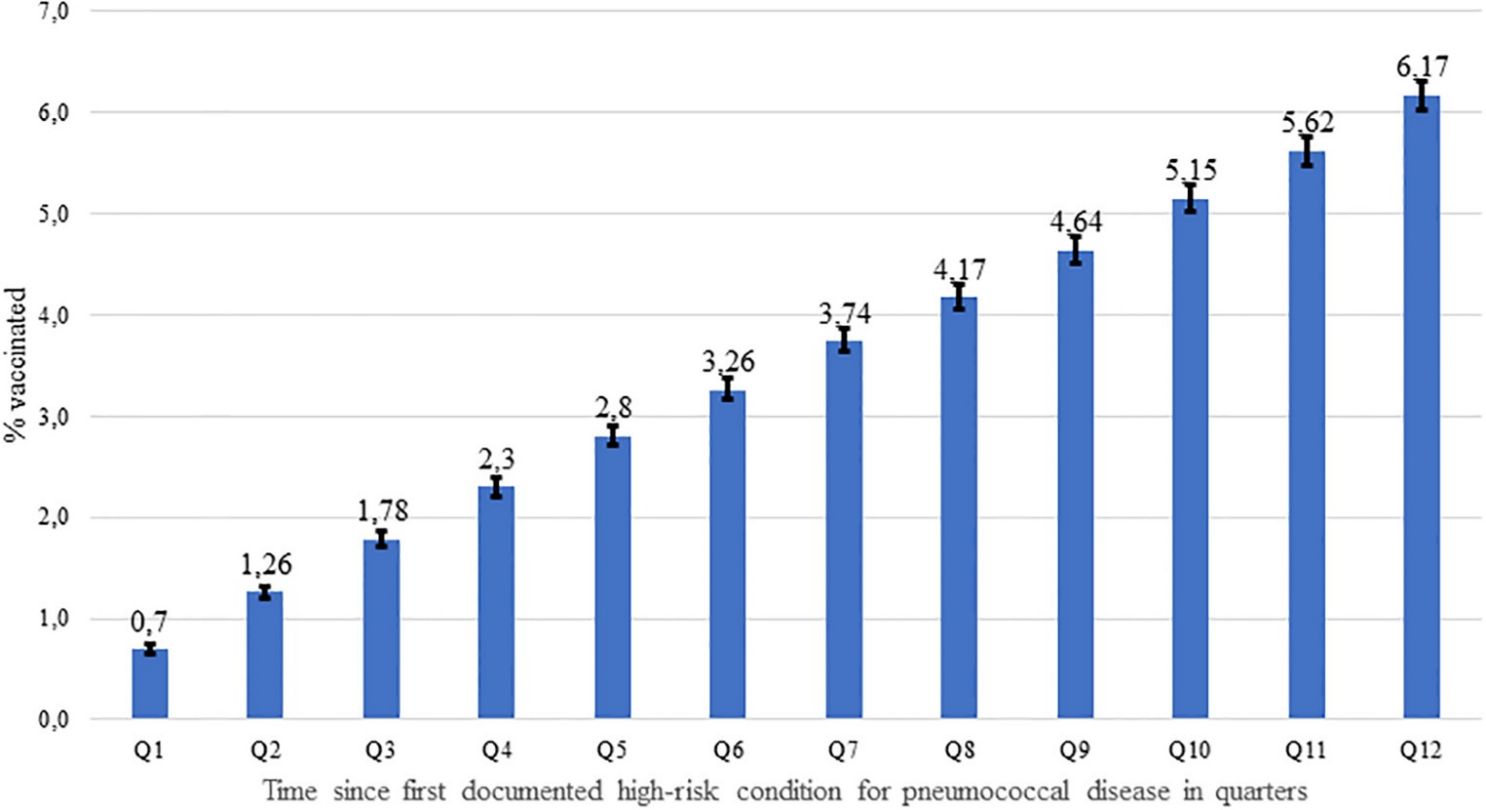
Cumulative pneumococcal vaccination rate within twelve quarters after first documented “high-risk” condition for pneumococcal disease.

The mean and median time to vaccination after the index date in 6,564 vaccinated patients within three years after the index date was 523 days (SD 329 days; 95% CI 515 days– 531 days) and 510 days (Q1 231 days, Q3 816 days), respectively. Similar to the primary analysis, the cumulative pneumococcal vaccination rate within three years after the index date increased constantly within the twelve quarters after the index date (Fig 3).

## Discussion

In this cohort study among 204,088 patients with a first episode of a “high-risk” condition for pneumococcal disease, the overall cumulative pneumococcal vaccination rate in Germany within two years and three years following the diagnosis was 4.4% and 6.2%, respectively. Similarly, the vaccination rate for specific “high-risk” conditions was low. Stratification by region revealed a slightly higher overall vaccination rate in Eastern Germany compared to Western Germany. The same trend was observed across age groups and all analyzed disease-specific subgroups. The median time to vaccination in vaccinated patients within the first two years after first documentation of a “high-risk” condition was high with 332.5 days. Patients were vaccinated in a constant manner over time and the majority of patients were vaccinated by a general practitioner (92.6%). Other medical specialists who are frequently involved in the diagnosis and management of patients with “high-risk” condition, e.g. rheumatologists, oncologists and pneumologists, only rarely administer vaccinations.

To our knowledge, our study is the first to document pneumococcal vaccination rates in incident high risk (i.e. immunocompromized) patients also reflecting the time to vaccination after initial diagnosis. A low vaccination rate after a first documented episode of a “high-risk” condition for pneumococcal disease according to the STIKO recommendations in Germany has also been observed in previous studies. Braeter et al. [9] investigated the cumulative pneumococcal vaccination rate with an individual 5-year follow-up of patients getting 60 years of age in 2010 based on ambulatory data from Germany between 2010 and 2014, and found a vaccination rate of 7.9% within 2 years and 15.7% within 5 years in patients with an incident “at-risk” or “high-risk” condition for pneumococcal disease. Interestingly, no differences were found between patients with incident “at-risk” condition and incident “high-risk” condition. Similar to our study, the vaccination rate was approximately twice as high in Eastern Germany compared to Western Germany and the lowest vaccination rates were observed in Southern Germany, i.e. Bavaria and Baden-Wuerttemberg. Theidel et al. [14] estimated the coverage for patients with underlying risk factors for pneumococcal disease of 5.9% to 12.7% in the age group 18–59 years and 34.5% to 54.7% in patients 60+ years in a simulation study based on data from German claims data between 2008 and 2009. Again, no significant differences were observed between patients with “at-risk” and “high-risk” condition for pneumococcal disease. In a survey based on data from an outpatient clinic among patients with prevalent rheumatoid arthritis, the overall coverage with pneumococcal vaccination was documented with 33% [11]. While this estimate is higher compared to our study, the study population included prevalent cases and did not focus on the two years after the incident diagnosis of rheumatoid arthritis as we did in our study. In another study performed 2013 in patients with rheumatoid arthritis based on German SHI claims data, a pneumococcal vaccination rate of 15.0% was found with similar regional trends, i.e. higher in Eastern Germany and lower in Southern federal states. Of note, event rates of pneumonia were higher in federal states with low pneumococcal vaccination coverage [10]. In a study within the framework of the Cooperative European Paediatric Renal Transplant Initiative, the pneumococcal vaccination coverage in 254 European children eligible for transplantation due to end-stage renal disease was also low with 42% [15].

Vaccination recommendation and funding are bound to have high impact on the vaccination rate since they influence physician’s decision-making process, economic impact for individual patients and coverage of vaccination damage. Until 2013, only PPSV23 was registered for prevention of pneumococcal diseases (PD) in adults in Germany, and as a consequence, recommended and reimbursed for elderly and patients at risk for PD. With the registration and availability of the 13-valent conjugate vaccine, the German reimbursement bodies reacted with a widening of reimbursement of both PPSV23 and PCV13 for adults at risk, giving the vaccinating physician a choice of vaccine to use. Since August 2016, STIKO recommends sequential pneumococcal vaccination with PCV13 followed by PPSV23 after 6–12 months [7] in patients with “high-risk” condition and children aged 2–15 years with “at-risk conditions”.

A general reason for the observed low pneumococcal vaccination rates could be the uncertainty regarding responsibility of vaccination between GPs and medical specialists in the outpatient setting, uncertainty with regard to the adequate time of vaccination as well as the fear to deteriorate the underlying “high-risk” condition. Our results highlight that further efforts to strengthen the awareness and improve the timeliness of pneumococcal vaccination among general practitioners and medical specialists, e.g. through standing recall-systems [16], could improve vaccination rates. In this context, a systematic documentation as provided by an electronic health record or an electronic vaccination card might improve the network among physicians and consequently increase the vaccination rate. Our study showed that “high-risk” patients are predominantly vaccinated by their GP, although physicians of all specialties may administer vaccines in Germany. Targeted education programs regarding pneumococcal vaccinations specifically conducted to those clinicians involved in the diagnosis and treatment of patients with “high-risk” conditions, i.e. specialists in internal medicine and subspecialists such as oncologists, rheumatologists or nephrologists, could further increase pneumococcal vaccination rates. For instance, vaccinations could be routinely administered at the time diagnosis of “high-risk” conditions or before start or treatment.

Pneumococcal vaccination uptake may also have been influenced by the controversial scientific debate with regard to the efficacy, effectiveness and serotype coverage of both vaccines available [17–22] prior to and after publication of current STIKO’s recommendation [23,24]. In the absence of studies with clinical outcomes, the evidence regarding the efficacy of PPSV23 and PCV13 in “high risk” patients was mainly based on immunogenicity studies. The recommendation for sequential vaccination with PCV13 followed by PPSV23 was based on the assumption that PPSV23 provides broader coverage of serotypes while PCV13 provides higher immunogenicity in patients with selected immunodeficiencies [24]. A systematic overview of the evidence is available in the scientific justification for the STIKO recommendation [24] and the German guideline for the management of adult community-acquired pneumonia and prevention [19]. The current differentiation of vaccination schemes between “at-risk” and “high-risk” patients appears difficult in daily routine, potentially leading to even reduced pneumococcal vaccination rates in the vulnerable group of “high-risk” patients since the publication of the vaccination recommendation in 2016. On the other hand, the scientific controversial scientific debate may have increased disease and vaccination awareness.

### Strengths and limitations

To our knowledge, this is the first study providing data on the pneumococcal vaccination rate, physician specialty of the vaccinating physicians and the timing of vaccination in patients with a first episode of a “high-risk” condition for pneumococcal disease in Germany. The main strength of our analysis is the high precision of estimates due to the large sample size of the underlying dataset obtained from the InGef database covering more than four million insured members of SHIs all over Germany, representative for the German population with regard to age and sex.

Although the database does not allow to differentiate between PCV13 and PPSV23 to report vaccination rates by vaccination type, it is well suitable for our research question on time of vaccination and vaccination rate in “high-risk” patients.

The study database has some limitations which we, however, assume to have no major impact on interpretation of our study results:

- Generalizability of pneumococcal vaccination rates and other outcomes obtained in this study may be limited, if differences exist by unknown or unobservable factors. For instance, data from persons insured in private health insurances may differ from statutory health insured persons due to diverging reimbursement practices or regarding their socioeconomic status; however, privately health insured population only reflects a minority of the total population of Germany with 10.6% (as of 2017) and therefore potential differences would only marginally affect our vaccination rate estimates. Furthermore, it needs to be kept in mind that our results cannot be directly extrapolated to other countries due to in part different health care systems and reimbursement practices.
- As our study did not include a review of individual patient files to confirm the presence of medical conditions, which for data protection reasons is generally not feasible, misclassification of “high-risk” conditions for pneumococcal disease according to STIKO cannot be ruled out. As a result, this may have led to an underestimation of the vaccination rate; however, we do not expect a major impact of on the interpretation of our results. Even in the unlikely scenario that half of our study population had been misclassified, the pneumococcal vaccination rate would have been below ten percent.
- Since the baseline period was limited to two years, left truncation in 2011 may have led to an underestimation of the pneumococcal vaccination rates, if patients had been vaccinated before, e.g. for other “at-risk” conditions. This is especially relevant for those individuals who might have received standard pneumococcal vaccination according to their age and not as part of a risk group, i.e. children who received PCV7 or PCV 13 as routine childhood immunization from 2006 onwards or patients age 60+ years. Therefore, we also analyzed “high-risk” patients in the age group 16–59 years for which the potential bias due to left truncation is assumed to be lowest, since the only alternative indication for vaccination pre baseline period would be an “at-risk” condition. Similar to all other age groups, we found a very low vaccination rate with 1.6% in women and 2.3% in men.
- Chronic renal failure and chronic severe liver disease, associated with immunosuppression, are defined as “high-risk” condition according to the current STIKO classification in our study but were defined as “at-risk” condition before August 2016. Since our study period covered the years from 2013 to 2016, our definition does not completely reflect the current STIKO guidelines during this time period. However, we do not expect a relevant effect on our results given that Theidel et al. [14] and Braeter et al. [9] did not find differences in the pneumococcal vaccination rates between patients “at-risk” and “high-risk” conditions. Furthermore, current STIKO recommendation also covers patients with cochlear implants or persons with cerebrospinal fluid leaks. Vaccination for this patient group has only been recommended as of September 2014 and reimbursed as of November 2014 and therefore could not be assessed in the underlying data. Since we focused our study on immune-compromised patients, this fact does not alter our results, however.
- The database only covers vaccinations in the outpatient setting. However, to our knowledge vaccination by default is not covered in the in-patient reimbursement system (DRG, disease related groups) in Germany and needs alternative reimbursement agreements. As a consequence, there is no incentive to vaccinate hospitalized patients and we assume inpatient vaccinations to be of minor extent with neglectable bias on our estimates of vaccination rate

## Conclusion

Our study suggests that the overall pneumococcal vaccination rate within two and three years after a new episode of a “high-risk” condition for pneumococcal disease is very low and that the vaccination after development of a “high-risk” condition is performed far too late. To prevent pneumococcal disease in patients at high-risk, further efforts to strengthen the awareness and improve the timeliness of pneumococcal vaccination in both general practitioners and medical specialists taking care of patients with high-risk condition according to STIKO are warranted.

## Supporting information

S1 Table Definition of variables. (DOCX)

S2 Table Pneumococcal vaccination rates with 95%-confidence intervals within two years in patients with “high-risk” condition for pneumococcal disease stratified by Association of Statutory Health Insurance Physicians. (DOCX)

S3 Table Mean and median time to vaccination in days within 2 years after incident “high-risk” condition stratified by AHIP. (DOCX)
