# Supplementary material for: Pneumococcal vaccination rates in immunocompromised patients—A cohort study based on claims data from more than 200,000 patients in Germany
Source: PLoS One. 2019 Aug 8;14(8):e0220848. doi: 10.1371/journal.pone.0220848 (PMC6687114; doi:10.1371/journal.pone.0220848)
Supplement: S1 Table — (DOCX) [file pone.0220848.s001.docx]

S1 Table Definition of variables

| **Variable** | **Role** | **Data source(s)** | **Operational definition** |
| --- | --- | --- | --- |
| Age | Baseline characteristic | Core data | Year of cohort entry – year of birth |
| Sex | Baseline characteristic | Core data | Sex in the year of cohort entry |
| Region | Subgroup | Core data | Region in the year of cohort entry classified according to the regional “Association of Statutory Health Insurance Physicians:   - Schleswig-Holstein - Hamburg - Bremen - Niedersachsen - Westfalen-Lippe - Nordrhein - Hessen - Rheinland-Pfalz - Baden-Wuerttemberg - Bayern - Berlin - Saarland - Mecklenburg-Vorpommern - Brandenburg - Sachsen-Anhalt - Thueringen - Sachsen |
| Functional or anatomic asplenia, sickle cell diseases and other hemoglobinopathies | Inclusion criteria / exclusion criteria  (high-risk condition for pneumococcal disease according to STIKO) | Ambulatory data, hospital data | Patients with a hospital diagnosis or verified or “status post” ambulatory diagnosis Q89.0, Q89.3, D56.*, D57.*, D58.2, D60.*, D61.*, D73.0, D73.1, D73.8  For hospital diagnoses, the admission date of the respective hospitalization was used as index date. For ambulatory diagnoses, the date of the first documented EBM-code by the diagnosing physician in the respective quarter was considered as index date. |
| Other immunodeficiency (incl. diseases of white blood cells) | Inclusion criteria / exclusion criteria  (high-risk condition for pneumococcal disease according to STIKO) | Ambulatory data, hospital data | Patients with a hospital diagnosis or verified ambulatory diagnosis (ICD-10 GM code) D55.*-D90.* (excl. D56*, D57*, D58.2, D60*, D61*, D73.0, D73.1, D73.8) |
| Malignant neoplasms excl. non-melanoma skin cancer | Inclusion criteria / exclusion criteria (subgroup)  (high-risk condition for pneumococcal disease according to STIKO) | Ambulatory data, hospital data, drug prescription data | Patients with a hospital diagnosis or verified ambulatory diagnosis (ICD-10 GM code) C00.*- C97.* (excl. C44.*), Z51.0, Z51.1 OR an OPS code 8542.*, 8543.*, 8544.0, 8544.1 (cytotoxic chemotherapy)  For hospital diagnoses, the admission date of the respective hospitalization was used as index date. For ambulatory diagnoses, the date of the first documented EBM-code by the diagnosing physician in the respective quarter was considered as index date. For OPS-codes the exact date was used as index date. |
| Stem cell transplantation | Inclusion criteria / exclusion criteria  (high-risk condition for pneumococcal disease according to STIKO) | Ambulatory data, hospital data | Patients with a hospital diagnosis or verified ambulatory diagnosis Z94.80, Z94.81 OR an OPS code 5411.*, 8805.*  For hospital diagnoses, the admission date of the respective hospitalization was used as index date. For ambulatory diagnoses, the date of the first documented EBM-code by the diagnosing physician in the respective quarter was considered as index date. For OPS-codes the exact date was used as index date. |
| HIV infection | Inclusion criteria / exclusion criteria (subgroup)  (high-risk condition for pneumococcal disease according to STIKO) | Ambulatory data, hospital data | Patients with a hospital diagnosis or verified ambulatory diagnosis (ICD-10 GM code) B20.*-B24.*, Z21  For hospital diagnoses, the admission date of the respective hospitalization was used as index date. For ambulatory diagnoses, the date of the first documented EBM-code by the diagnosing physician in the respective quarter was considered as index date. For OPS-codes the exact date was used as index date. |
| Chronic renal failure | Inclusion criteria / exclusion criteria (subgroup)  (high-risk condition for pneumococcal disease according to STIKO) | Ambulatory data, hospital data | Patients with a hospital diagnosis or verified ambulatory diagnosis (ICD-10 GM code) I12.*, I13.*, N00.*, N01.*, N03.*, N04.*, N05.*, N18.*, N19.*, Q60.*, Z49.*, Z94.0, Z99.2 OR an OPS code 8853.*, 8854.*, 8855.*, 8857.* (dialysis) OR EBM code 13602, 13610, 13611, 40823-40828 (dialysis)  For hospital diagnoses, the admission date of the respective hospitalization was used as index date. For ambulatory diagnoses, the date of the first documented EBM-code by the diagnosing physician in the respective quarter was considered as index date. For OPS-codes and EBM-codes, the exact date was used as index date. |
| Chronic severe liver disease | Inclusion criteria / exclusion criteria  (high-risk condition for pneumococcal disease according to STIKO) | Ambulatory data, hospital data | Patients with a hospital diagnosis or verified ambulatory diagnosis (ICD-10 GM code) K70.* (excl. K70.0), K71.3-K71.8, K72.1-K72.9, K73.*, K74.*, K75.0, K75.1, K76.2-7, I85.0, I85.9  For hospital diagnoses, the admission date of the respective hospitalization was used as index date. For ambulatory diagnoses, the date of the first documented EBM-code by the diagnosing physician in the respective quarter was considered as index date. |
| Immunosuppressant use | Inclusion criteria / exclusion criteria  (high-risk condition for pneumococcal disease according to STIKO) | Drug prescription data | Patients with a prescription of a drug with the ATC code L04* and M01CX01 (methotrexate)  The index date was 30 days prior to treatment initiation (prescription date) |
| Chronic heart disease | Inclusion criteria / exclusion criteria (in patients aged 2-15 years only)  (at-risk condition for pneumococcal disease according to STIKO) | Ambulatory data, hospital data | Patients with a hospital diagnosis or verified ambulatory diagnosis (ICD-10 GM code) I05.*-I09.*, I11.*, I13.*, I20.*, I21.*, I22.*, I24.*, I25.1, I25.2, I27.*, I34.*-I39.*, I42.*, I50.*, I23.*, Q20.*-Q24.*, Q25.1  For hospital diagnoses, the admission date of the respective hospitalization was used as index date. For ambulatory diagnoses, the date of the first documented EBM-code by the diagnosing physician in the respective quarter was considered as index date. |
| Chronic pulmonary disease (incl. asthma) | Inclusion criteria / exclusion criteria (in patients aged 2-15 years only)  (at-risk condition for pneumococcal disease according to STIKO) | Ambulatory data, hospital data | Patients with a hospital diagnosis or verified ambulatory diagnosis (ICD-10 GM code) E84.*, I27.8, I27.9, J40.*-J47.*, J60.*-J65.*, J68.4, J84.*, J96.*, P27.*  For hospital diagnoses, the admission date of the respective hospitalization was used as index date. For ambulatory diagnoses, the date of the first documented EBM-code by the diagnosing physician in the respective quarter was considered as index date. |
| Diabetes treated with oral antidiabetics or insulin | Inclusion criteria / exclusion criteria (in patients aged 2-15 years only)  (at-risk condition for pneumococcal disease according to STIKO) | Ambulatory data, hospital data, drug prescription data | Patients with a hospital diagnosis or verified ambulatory diagnosis (ICD-10 GM code) E10.*-E14.*, G59.0, G63.2, P70.1, T38.3, AND prescription of a drug with the ATC-code A10A*, A10B*  For hospital diagnoses, the admission date of the respective hospitalization was used as index date. For patients with an ambulatory diagnosis, the date of the prescription of an oral antidiabetic or insulin in respective quarter was used as the index date |
| Neurological disorders | Inclusion criteria / exclusion criteria (in patients aged 2-15 years only)  (at-risk condition for pneumococcal disease according to STIKO) | Ambulatory data, hospital data | Patients with a hospital diagnosis or verified ambulatory diagnosis (ICD-10 GM code) G10.*, G11.*, G12.*, G13.*, G20.*, G21.*, G22.*, G23.*, G24.*, G25.*, G26.*, G30.*, G31.*, G32.*, G35.*, G37.*, G40.*, G41.*, G70.*, G71.*, G72.*, G73.*, G80.*, G81.*, G82.*, F00.*-F03.*, F05.1, G30.*, G31.1, I60.*-I64, P90, R56  For hospital diagnoses, the admission date of the respective hospitalization was used as index date. For ambulatory diagnoses, the date of the first documented EBM-code by the diagnosing physician in the respective quarter was considered as index date. |
| Rheumatoid arthritis | Subgroup in combination with immunosuppressant use or not | Ambulatory data, hospital data | Patients with a hospital diagnosis or verified ambulatory diagnosis (ICD-10 GM code) M05.*, M06.*  For hospital diagnoses, the admission date of the respective hospitalization was used as index date. For ambulatory diagnoses, the date of the first documented EBM-code by the diagnosing physician in the respective quarter was considered as index date. |
| Specialty of the vaccinating physician | Outcome | Ambulatory data | Specialty of the vaccinating physician will classified into the following groups:   - General practitioner - Rheumatologist - Oncologist - Pneumologist - Other internist - Pediatrician - Other specialty - Unknown |
| Pneumococcal vaccination | Outcome | Ambulatory data | Patients with an EBM code 89118, 89119, 89120  Vaccinations were assessed on the exact date of documented EBM-code |
