# Supplementary material for: Pneumococcal vaccination rates in immunocompromised patients—A cohort study based on claims data from more than 200,000 patients in Germany
Source: PLoS One. 2019 Aug 8;14(8):e0220848. doi: 10.1371/journal.pone.0220848 (PMC6687114; doi:10.1371/journal.pone.0220848)
Supplement: S2 Table — (DOCX) [file pone.0220848.s002.docx]

S2 Table Pneumococcal vaccination rates with 95%-confidence intervals within two years in patients with “high-risk” condition for pneumococcal disease stratified by AHIP

|  | **N cohort** | **N vaccinated** | **Vaccination rate within 2 years after index date in % (95%-CI)** |
| --- | --- | --- | --- |
| **Overall** | 204,088 | 8,892 | 4.4 (4.3-4.5) |
| **Association of statutory health insurance physician (AHIP)** |  |  |  |
| Schleswig-Holstein | 5,843 | 280 | 4.8 (4.3-5.4) |
| Hamburg | 2,046 | 97 | 4.4 (4.3-4.5) |
| Bremen | 793 | 21 | 2.7 (1.7-4.0) |
| Niedersachsen | 22,397 | 1,127 | 5.0 (4.8-5.3) |
| Westfalen-Lippe | 29,432 | 1,294 | 4.4 (4.2-4.6) |
| Nordrhein | 36,354 | 1,754 | 4.8 (4.6-5.1) |
| Hessen | 15,164 | 614 | 4.1 (3.7-4.4) |
| Rheinland-Pfalz | 15,396 | 609 | 4.0 (3.7-4.3) |
| Baden-Wuerttemberg | 26,917 | 959 | 3.6 (3.3-3.8) |
| Bayern | 32,578 | 1,132 | 3.5 (3.3-3.7) |
| Berlin | 2,914 | 143 | 4.9 (4.2-5.8) |
| Saarland | 1,745 | 67 | 3.8 (3.0-4.9) |
| Mecklenburg-Vorpommern | 3,256 | 233 | 7.2 (6.3-8.1) |
| Brandenburg | 2,834 | 176 | 6.2 (5.4-7.2) |
| Sachsen-Anhalt | 1,591 | 83 | 5.2 (4.2-6.4) |
| Thueringen | 2,541 | 141 | 5.6 (4.7-6.5) |
| Sachsen | 2,046 | 158 | 7.7 (6.6-9.0) |

CI = Confidence interval
