# Supplementary material for: Pneumococcal vaccination rates in immunocompromised patients—A cohort study based on claims data from more than 200,000 patients in Germany
Source: PLoS One. 2019 Aug 8;14(8):e0220848. doi: 10.1371/journal.pone.0220848 (PMC6687114; doi:10.1371/journal.pone.0220848)
Supplement: S3 Table — (DOCX) [file pone.0220848.s003.docx]

S3 Table Mean and median time to vaccination in days within 2 years after incident “high-risk” condition stratified by AHIP

|  | **Mean (SD; 95%-CI)** | **Median (Q1; Q3)** |
| --- | --- | --- |
| **Overall** | 340 (219; 336 to 345) | 332.5 (142; 528) |
| **Association of statutory health insurance physician** |  |  |
| Schleswig-Holstein | 349 (218; 323to 374) | 346 (148; 538) |
| Hamburg | 315 (214; 272 to 358) | 295 (113; 496 |
| Bremen | 312 (217; 214 to 411) | 315 (134; 402 |
| Niedersachsen | 345 (224; 332 to 358) | 346 (138; 538) |
| Westfalen-Lippe | 340 (223; 328 to 352) | 331 (135; 538) |
| Nordrhein | 341 (215; 331 to 351) | 332.5 (150; 517) |
| Hessen | 349 (222; 331 to 367) | 342 (144; 550) |
| Rheinland-Pfalz | 319 (223; 301 to 336) | 302 (112; 510) |
| Baden-Wuerttemberg | 339 (218; 325 to 353) | 336 (137; 525) |
| Bayern | 340 (219; 328 to 353) | 328 (150.5; 531) |
| Berlin | 324 (213; 289 to 359) | 313 (120; 504) |
| Saarland | 337 (221; 283 to 391) | 365 (112; 555) |
| Mecklenburg-Vorpommern | 336 (220; 307 to 364) | 315 (149; 536) |
| Brandenburg | 339 (213; 307 to 370) | 337.5 (158; 509) |
| Sachsen-Anhalt | 337 (214; 290 to 384) | 338 (149; 509) |
| Thueringen | 376 (220; 339 to 413) | 367 (179; 572) |
| Sachsen | 366 (211; 333 to 400) | 369.5 (184; 548) |

CI = Confidence interval; SD = Standard deviation
